# Optimizing Agricultural Input and Production for Different Types of at-Risk Peasant Households: An Empirical Study of Typical Counties in the Yimeng Mountain Area of Northern China

**DOI:** 10.3390/ijerph192113938

**Published:** 2022-10-26

**Authors:** Yuanhe Yu, Liang Wang, Jinkuo Lin, Zijun Li

**Affiliations:** 1 College of Geography and Environment, Shandong Normal University, Jinan 250358, China; 2 School of Environment and Natural Resources, Renmin University of China, Beijing 100872, China; 3 Shandong Provincial Key Laboratory of Water and Soil Conservation and Environmental Protection, Linyi University, Linyi 276000, China

**Keywords:** peasant household, risk state, agricultural input, production combination, optimization, target MOTAD model

## Abstract

Using typical counties in the Yimeng Mountain area of northern China as an example, this paper analyzed the household and agricultural input characteristics of different types of peasant households using survey data from 262 farm households. The target minimization of the total absolute deviations (MOTAD) model was applied to determine the optimal combinations in the allocation of agricultural input factors and production for different types of at-risk peasant households to obtain the ideal agricultural income. The relevant results are twofold. (1) The agricultural input behaviors of different types of peasant households vary significantly. The highest levels of agricultural land, labor, and yield-increasing and labor-saving inputs included I part-time peasant households (*I PTPH*), followed by full-time peasant households (*FTPH*), while the input levels of II part-time peasant households (*II PTPH*) and non-agricultural peasant households (*NAPH*) with higher levels of non-agricultural employment gradually decreased. In general, an increase in peasant households’ part-time employment revealed an inverted U-shaped trend in the agricultural input level, with a trajectory of *I PTPH* > *FTPH* > *II PTPH* > *NAPH*. (2) The current agricultural inputs and production combinations of different types of peasant households have room for improvement. It is necessary to adjust agricultural inputs and optimize production combinations to obtain target incomes. Overall, all types of peasant households must streamline labor inputs and increase capital inputs, except for *I PTPH*, for which capital inputs should be reduced. Following optimization, economic crops gradually replace grain crops, and the optimal agricultural incomes of peasant households will be improved. The study results provide practical policy insights for reducing agricultural production risks and improving agricultural production incomes.

## 1. Introduction

Agriculture is essentially a typical risk activity, and contemporary global warming and population growth pose unprecedented challenges for agriculture [1]. Some developing countries are already facing food crises, which seriously threaten national security and social stability and hinders the sustainable development goals of the United Nations [2,3]. Like many developing countries in the world, China also faces challenges arising from agricultural risks. The agriculture risks in China are complex, and the losses are considerable [4,5]. According to the report on natural disasters in China in 2019, released by the Ministry of Emergency Management, 19,260 thousand hectares of agricultural area was affected in China, translating to a direct economic loss of 327.09 billion CNY. In addition, market, policy, financial, technology, and other risks cross-fertilize one another [6], making agricultural production and operation face great instability [7,8,9].

Many studies have confirmed that the rise in agricultural risk is attributable to a variety of factors, such as climate change, natural disasters, geographic conditions, imperfect markets, changes in agricultural yields and prices, and the lack of finance, credit, and insurance [10,11,12,13,14,15]. As the fundamental organizational unit of agricultural production and livelihood, peasant households are highly vulnerable to agricultural risks. When facing risks, the most vulnerable groups are often peasant households with insufficient resources and poor adaptability [16]. For example, climate change risks can seriously affect ecosystem services and agricultural production, increasing food insecurity for peasant households [11,17]. Financial risks can raise the cost of credit and hinder the diversification of peasant households’ livelihood activities, limiting the potential to increase peasant households’ income [18]. Social risks can reduce opportunities for peasant households to participate in off-farm activities [6,19]. Many studies have demonstrated that appropriate production adaptation decisions are an effective way for peasant households to cope with external shocks and risks [20,21,22]. Therefore, in the context of the increasing risks faced by peasant households, it is significant for such households in developing countries to understand the potential adaptive agricultural production decisions to effectively mitigate and neutralize agricultural risks.

As micro agents of agricultural production in China, the extent to which peasant households respond to agricultural risks is critical for making agricultural production and investment decisions. Peasant households’ risk preferences may influence the choice and scale of production projects, further affecting the micro-agricultural production structure and agricultural income [23]. If peasant households have a risk appetite, they will tend to choose high-risk and potentially high-return production projects in their production decisions, whereas if they are risk averse, they will be reluctant to pursue new measures because of the uncertainty of the costs and returns [15,24]. Numerous studies have shown that farm size, geographic location, land ownership, household income, age, and education highly influence the extent to which peasant households respond to agricultural risks, with a significant impact on peasant households’ production decisions [10,25,26,27,28]. The issue of risk decision making in agricultural production has long been a topic of interest in agricultural economics, and a great deal of fruitful research has been conducted. Many scholars have pointed out that most peasant households are risk averse by nature, which is more prominent among small peasant households [29]. Peasant households, whose production efficiency is suboptimal in a risk state, tend to reduce agricultural risk by making production decisions, such as crop diversification [24,30,31,32,33], planting superior varieties [16,34], crop rotation or intercropping [35,36,37], and developing mixed agriculture, combining agriculture and animal husbandry [9,38]. In addition, peasant households employ other livelihood strategies to mitigate the effects of agricultural risks, such as employment diversification, consumption reduction, agricultural input adjustment, migration, insurance, and credit [22,39,40,41]. It is evident that agricultural production decisions in risk states are critical to risk avoidance and impact the optimal combination of risk and return, resulting in a wide range of outcomes.

In summary, many scholars have conducted extensive research on the degree of peasant households’ response to agricultural risks, the influencing factors of agricultural risks, and how to deal with agricultural risks. Previous studies have established a solid foundation for examining peasant households’ production decision-making behavior under risk states; however, most research objects have been individual farms or peasant households in different regions. With the gradual development of the rural economy, peasant households’ internal differentiation has become more prominent [42,43]. The variations in response to agricultural risks among different types of peasant households are evident, and households must constantly adjust agricultural production decisions to ensure their own survival and meet the needs of the market economy to achieve optimal production efficiency. In practice, any production decision made by different types of peasant households involves certain risks [44], and different types of peasant households often adjust the agricultural input and optimize an agricultural production combination according to their resource endowment conditions, expected returns, and risk tolerance. Existing studies have paid little attention to the agricultural production decisions of different types of peasant households in at-risk states, and even less research has been conducted on the optimization of agricultural input behavior in peasant households’ agricultural production processes [21,45]. Based on this, this paper selected the Yimeng Mountain area in northern China as the study area, examining survey data from peasant households in two typical counties to systematically analyze the differences in the agricultural input behavior of different types of peasant households. The target minimization of the total absolute deviations (MOTAD) model was used to explore the potential adjustment and optimization of the combination of agricultural inputs and production for different types of at-risk peasant households to provide scientific references for improving the efficiency of agricultural land resource use and increasing peasant households’ income.

The remainder of this study is structured as follows. Section 2 presents an overview of the Yimeng Mountain area, data sources, and research methods. Section 3 compares the household and agricultural input characteristics of different types of peasant households and analyzes the process of agricultural input factors allocation and production combination optimization for different types of at-risk peasant households. Section 4 details and compares the results, and Section 5 concludes the study, proposing relevant solutions and countermeasures.

## 2. Materials and Methods

### 2.1. Study Area

**Figure 1:**
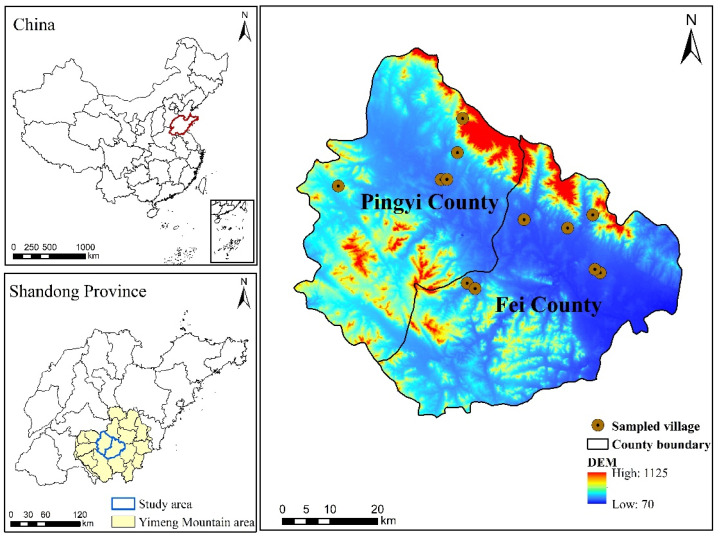
Geographic location of the study area.

The Yimeng Mountain area is located in southern Shandong Province, China, at latitude 34°23′–36°18′ N and longitude 116°40′–119°11′ E, with a total area of about 28,804.5 km^2^ (Figure 1). Landform types are complex and diverse, among which mountains and hills account for about 70% of the total area. The climate type is a warm-temperate continental monsoon climate, with an average annual temperature of 13–14.3 °C and an average annual rainfall of 815 mm. The region is rich in natural resources, and agriculture occupies a large proportion of the economic development; however, due to the poor soil and the large expanse of sloping arable land, the level of agricultural development is relatively backward. Pingyi County and Fei County are located in the core of the Yimeng Mountain area, of which Pingyi County had a total area of about 1822.95 km^2^, with 555 administrative villages in 14 towns, a gross agricultural product of 5.006 billion CNY, and 496,400 rural employees in 2020; Fei County had a total area of about 1659.9 km^2^, 411 administrative villages in 12 towns, a gross agricultural product of 4.881 billion CNY, and 439,400 rural employees. Both counties are large mountainous agricultural counties, and agriculture has a dominant place in the region’s economic development. Land-use types in both counties are primarily crop and garden land. Crops predominantly include wheat, corn, and other grain crops as well as economic crops, such as peanuts, honeysuckle, and garlic, while forest and fruit crops mainly include chestnuts and peaches. Agricultural labor emigration is common, and peasant households’ part-time employment behavior is prominent, giving rise to different types of peasant households, which is more representative for investigating the optimization of combination of agricultural inputs and production for different types of peasant households.

### 2.2. Data Sources

This study mainly acquired data from a questionnaire survey of peasant households conducted by the research group in Pingyi and Fei counties of Shandong Province from September to October 2018. The questionnaire included five aspects: peasant household members’ basic information, agricultural input and output, peasant households’ income and expenditure, agricultural land transfer, and planting intentions. The questionnaire survey was conducted in 12 administrative villages in six townships, distributing 265 questionnaires and obtaining 252 valid questionnaires (113 in Pingyi County and 139 in Fei County), with a 95.1% response rate. The data on peasant households’ agricultural returns were limited in the survey and do not fully reflect the fluctuation of agricultural product income. To overcome this, we used data regarding the major agricultural returns in Shandong Province from the National Compilation of Agricultural Product Costs and Returns (2013–2017) to represent the available risk information for peasant households according to fluctuation status, and the data regarding the fluctuation of major agricultural returns in these five years were used as the basic information to simulate the risk decision-making behavior of peasant households’ agricultural production and operations.

### 2.3. Research Methods

#### 2.3.1. Classification of Peasant Household Types

There are various methods for classifying peasant households, most of which do not include peasant households’ non-labor income other than agricultural and non-agricultural income, such as national policy subsidies, regional welfare subsidies, pensions, retirement wages, and child support. Considering the actual circumstances in the rural regions in the Yimeng Mountain area and drawing on the results of existing studies [46,47], this study classified peasant households into four types: full-time peasant household (*FTPH*), I part-time peasant household (*I PTPH*), II part-time peasant household (*II PTPH*), and non-agricultural peasant household (*NAPH*), using households’ main earning activities, the allocation of agricultural and sideline products, and the main sources and structures of household income as the criteria for classifying peasant households (Table 1).

#### 2.3.2. Target MOTAD Model

The Target MOTAD model is an improvement on the original MOTAD model, which has the advantages of not requiring strict assumptions regarding the utility function of peasant households’ agricultural production or knowledge of peasant households’ risk tolerance [24,33]. In addition, it can be assumed that the utility function increases for income and decreases for risk, which has strong applicability and has been widely used in the analyses of agricultural operation risk [48,49,50]. This study employed the Target MOTAD model to analyze the optimal combinations of agricultural input factor allocation and production for different types of at-risk peasant households. The Target MOTAD model constructed in this study is as follows:

{(1)MaxE(z)=∑j=1ncjxj(2)s.t.∑j=1nakjxj≤bk  (k=1,⋯,m)(3)∑j=1ncrjxj−yr≥T  (r=1,⋯,s)(4)∑r=1spryr=λ  (λ=M→0)

Equation (1) is the objective function, where *E*(*z*) denotes peasant households’ expected income, *c_j_* denotes the expected income per unit area of peasant households’ *j_th_* agricultural production, and *x_j_* denotes the scale of peasant households’ *j_th_* agricultural production.

Equation (2) estimates peasant households’ resource endowment constraint, where *a_kj_* denotes the demand per unit area of the *k_th_* resource for the peasant household’s *j_th_* agricultural production activity, *b_k_* denotes the total amount of the *k_th_* resource owned by a peasant household, and *m* denotes the types of resources owned by a peasant household.

Equation (3) represents the deviation between expected income and the given target income of the peasant household under risk state *r*, where *c_rj_* represents the expected income per unit area of the peasant household’s *j_th_* agricultural production in the risk state, *y_r_* represents the deviation of the peasant household’s actual income from the target income, *T* represents the peasant household’s target income, and *s* represents the number of cycles (i.e., years of agricultural production by the sample at-risk peasant household, and *s* = 5 in this study).

Equation (4) is the crucial part of the model, which indicates the strength of the risk faced by the peasant household, where *p_r_* represents the probability of risk state *r* occurring (*p_r_* = 1/s), *λ* represents the at-risk value of the peasant household’s production combination in the range of [0, *M*], and *M* is a positive number greater than 0.

To reflect the relationship between the peasant households’ risks and target incomes more clearly, the following equation is obtained by substituting Equation (3) into Equation (4):

(5) $$\begin{array}{l} \lambda =\sum_{r=1}^{s}p_{r}\cdot \left\{ \begin{array}{l} \left(T-\sum_{j=1}^{n}c_{rj}x_{j}\right),T-\sum_{j=1}^{n}c_{rj}x_{j}\;\geq \;0\\ 0,T-\sum_{j=1}^{n}c_{rj}x_{j}<0 \end{array}\right. \end{array}$$

In Equation (5), when $T-\sum_{j=1}^{n}c_{rj}x_{j}\geq 0$, the risk faced by peasant households in agricultural production activities is an increasing function of the deviation between the target and actual incomes. The maximum value of *λ* is the maximum income for a given target income (*T*). Under a given target income (*T*), as the value of *λ* gradually decreases, the risks faced by peasant households in agricultural production also gradually decrease, establishing the optimal change in the production combination. Under the given optimal production combination, the optimal allocation structure of agricultural input factors can be achieved to optimize the agricultural input behavior of peasant households. Referring to the related research results [8,10,18,31,50], the selected variables and their description are shown in Table 2.

## 3. Results

### 3.1. Statistical Analysis of Peasant Household Types

**Figure 2:**
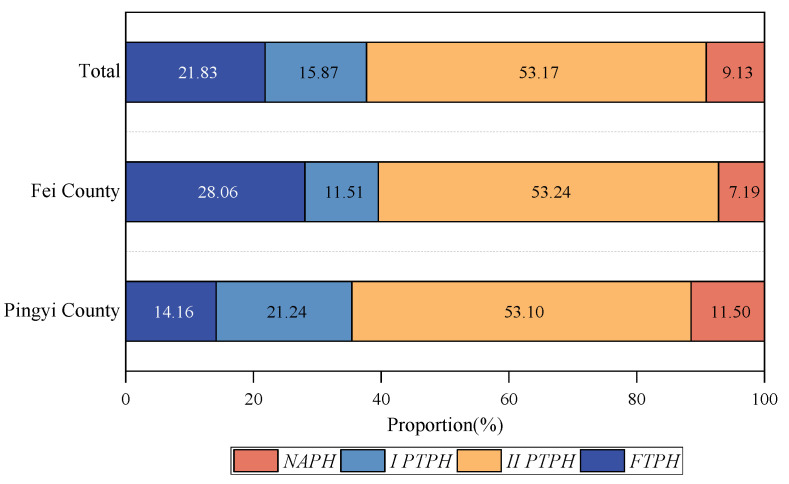
Results of the classification of peasant household types.

According to the classification criteria in Table 1, among the total sample of peasant households in the study area, 55 were *FTPH*, accounting for 21.83% of the total sample; 40 were *I PTPH*, accounting for 15.87% of the total sample; 134 were *II PTPH*, accounting for 53.17% of the total sample; and 23 were *NAPH*, accounting for 9.13% of the total sample. Part-time peasant households exceeded 69% of the total sample, indicating that peasant households’ part-time employment behavior was more prominent in the study area. By county, the highest proportion of *II PTPH* in Pingyi County and Fei County was 53.10 and 53.24%, respectively. The highest proportion of *FTPH* was in Fei County, at 28.06%; the highest proportion of *I PTPH* was in Pingyi County, at 21.24%; and the lowest proportion of *NAPH* was in the Pingyi and Fei counties, at 11.50 and 7.19%, respectively (Figure 2).

### 3.2. Basic Household Characteristics of Different Types of Peasant Households

Table 3 reveals significant differences in the resource endowments of the different types of peasant households. In terms of household size, the *I PTPH*, *II PTPH*, and *NAPH* all exceeded four individuals, and the *NAPH* had the most, at 4.3 individuals. In addition, the household labor force of the *I PTPH*, *II PTPH*, and *NAPH* was greater than three individuals, and the highest was the *NAPH* at 3.39 individuals. In contrast, the household size and labor force of the *FTPH* were relatively small, at 2.38 and 1.93 individuals, respectively, much lower than the other three types of peasant households. However, the overall number of agricultural laborers in each type of peasant household was relatively close to about two. Among them, the number of agricultural laborers in the *FTPH* equaled that of household laborers. In contrast, the number of agricultural laborers in the *I PTPH*, *II PTPH*, and *NAPH* were all lower than the household labor force, which was consistent with the part-time employment behavior of laborers in the study area.

Regarding the age of the agricultural labor force, all types of peasant households were over 50 years old, among which the highest was 58.68 years old for the *FTPH*. In terms of the literacy level of the agricultural labor force, all types of peasant households were below three (i.e., the average literacy level was less than junior school), among which the highest literacy level was 2.61 for the *I PTPH*, while the lowest literacy level was 1.71 for the *NAPH*, indicating that the phenomenon of “old age and low culture” is prevalent in agricultural production in the study area.

In terms of the agricultural land resource endowment, the per capita agricultural land area of all types of peasant households was less than 2 mu, among which the *FTPH* had the most, at 1.61 mu. In contrast, the per capita agricultural land area gradually decreases as the degree of peasant households’ part-time employment increases, with 1.19, 0.78, and 0.54 mu for the *I PTPH*, *II PTPH*, and *NAPH*, respectively, reflecting a lack of agricultural land resources in the study area.

Regarding the household income, significant differences were evident in the income sources and income structures of the various types of peasant households. In terms of the total household income and non-agricultural income, the *NAPH* was the highest, with 57,670.35 and 54,869.57 CNY/year, respectively, followed by the *II PTPH* and *I PTPH* at a lower level, and the *FTPH* at the lowest, with almost no non-agricultural income. In terms of agricultural income, the *I PTPH* was the highest, at 19,748.30 CNY/year, and the *FTPH* was the second highest, at 13,109.11 CNY/year, while the *II PTPH* and *NAPH* were lower, at 6628.85 and 2695.57 CNY/year, respectively. In terms of non-labor income, the *FTPH* was the highest, at 1389.73 CNY/year, while all the other types of peasant households were lower. In terms of the household income structure, there were significant differences among the *FTPH*, *I PTPH*, *II PTPH*, and *NAPH*, with the proportion of agricultural income to total household income at 90.41, 51.87, 16.08, and 4.67%, respectively. Clearly, agriculture has a prominent role in peasant households’ production and operation activities in the study area.

Significant differences were evident in the level and structure of expenditures in the various types of peasant households. The *NAPH* had the highest total household expenditure of 25,273.67 CNY/year, of which the vast majority was allocated to living expenses, accounting for about 93.24%, while the agricultural production expenses were only 6.76% (1708.45 CNY/year). The total household expenditure of the *I PTPH* also reached 25,054.64 CNY/year, most of which was living expenses, accounting for about 65.18%, and the agricultural production expenditure accounted for about 34.82%. The total household expenditure of the *II PTPH* was 22,097.62 CNY/year, with an expenditure structure similar to that of *NAPH*, with the agricultural production expenditure and living expenses accounting for 14.94 and 85.06%, respectively. The total household expenditure of the *FTPH* was the lowest, at 12,655.50 CNY/year, and the proportions of the agricultural production and living expenses were closer, at 47.95 and 52.05%, respectively.

### 3.3. Agricultural Input Characteristics of Different Types of Peasant Households

The peasant households’ inputs in the agricultural production process primarily include agricultural land, labor, and capital (Table 4). In this study, the agricultural land input is the peasant households’ agricultural land area, the labor input is the households’ labor input time per unit area of agricultural land, and the total labor time input is the product of the labor input time per unit area of agricultural land and the peasant households’ total agricultural land area. The survey established that the sample peasant households rarely hired labor, so hired labor was not counted within the labor input but was converted to capital input according to the price of hired labor. Capital input mainly refers to the material and service costs spent by peasant households on seeds, pesticides, fertilizers, agricultural films, machinery power, and other needs per unit area of agricultural land. According to the purpose of capital input, it is divided into yield-increasing input and labor-saving input. For example, seeds, fertilizers, pesticides, agricultural films, and other needs are used to increase the yield of agricultural land and are yield-increasing inputs, whereas mechanical inputs and herbicides replace labor inputs as labor-saving inputs.

In terms of the agricultural land input, the sample peasant households’ average agricultural land area was 3.39 mu, and the agricultural land scale was generally small. The trajectory of the agricultural land scale among different types of peasant households was *I PTPH* (4.95 mu) > *FTPH* (3.84 mu) > *II PTPH* (3.32 mu) > *NAPH* (2.33 mu), indicating that the increase in the part-time employment level had a trend of first rising and then declining. The average number of sample peasant households’ agricultural land plots was close to five pieces, indicating that the fragmentation of agricultural land in the study area was relatively severe, among which the *I PTPH* had the most, at 8.6 pieces, and the *II PTPH*, *NAPH*, and *FTPH* did not differ much, at 4.77, 4.38, and 4.20 pieces, respectively.

The average labor input per unit area of the sample peasant households’ agricultural land was 53.55 workdays/mu, and the different types of peasant households’ labor input per unit area showed a trajectory of *FTPH* (75.02 workdays/mu) > *I PTPH* (63.35 workdays/mu) > *II PTPH* (55.87 workdays/mu) > *NAPH* (44.49 workdays/mu), indicating that as the level of peasant households’ part-time employment increased, the labor input per unit area of agricultural land had a significant decreasing trend. However, the total labor input revealed a trajectory of *I PTPH* (313.57 workdays) > *FTPH* (296.32 workdays) > *II PTPH* (185.50 workdays) > *NAPH* (103.66 workdays), which was primarily related to the *I PTPH* having the largest area of agricultural land.

The sum of the yield-increasing capital input per unit area was much higher than the labor-saving input per unit area for the different types of peasant households, but significant differences were evident. Among the yield-increasing inputs, the fertilizer input per unit area was the highest, with an average of 317.86 CNY/mu, of which the *I PTPH* was the highest, at 482.24 CNY/mu; the *FTPH* was the second highest, at 380.37 CNY/mu; the *II PTPH* was lower; and the *NAPH* was the lowest. The seed input per unit area was also high, with an average input of 140.07 CNY/mu, of which the highest was the *FTPH*, at 220.96 CNY/mu, and the lowest was the *NAPH*, at 85.35 CNY/mu. The pesticide input per unit area averaged 98.98 CNY/mu, among which the highest was 318.66 CNY/mu for the *I PTPH*, the second highest was 161.94 CNY/mu for the *FTPH*, and the lowest was 42.63 CNY/mu for the *NAPH*. The agricultural film was the least yield-increasing input, with an average input of 27.06 CNY/mu, among which the highest was 44.57 CNY/mu for the *FTPH*. In terms of the labor-saving input, the average was 118.70 CNY/mu, which was lower than the input level of seeds and fertilizers, among which the highest was 167.61 CNY/mu for the *I PTPH*, indicating a deficit in the overall agricultural mechanization level in the study area, likely due to factors such as mountainous land and fragmented plots.

The average total capital input in the study area was 4055.91 CNY, and the level was not high. The total capital input differed among the different types of peasant households, showing an inverted U-shaped pattern of rising first and then decreasing as the level of part-time employment rose. Among them, the *I PTPH* was the highest, at 8724.64 CNY; the *FTPH* was second, at 6068.22 CNY; and the *II PTPH* and *NAPH* were lower, at 3300.86 and 1708.45 CNY, respectively.

Overall, significant differences in the agricultural land, labor, and capital inputs were evident among the different types of peasant households in the study area, which were closely related to the business objectives and production decisions of the different types of at-risk peasant households. For the peasant households, the main business objective is simultaneously allocating the appropriate agricultural inputs under risk states, improving the agricultural production efficiency, and increasing the agricultural income. Consequently, it is necessary to further explore the optimal allocation of the agricultural input factors and production combinations for the different types of at-risk peasant households to provide a practical reference for reducing agricultural risks and increasing farmers’ income.

### 3.4. Optimizing the Allocation of Agricultural Input Factors and Production Combinations for Different Types of at-Risk Peasant Households

#### 3.4.1. Target MOTAD Model

##### Planting System Constraints

Both Pingyi County and Fei County are located in the Yimeng Mountain, with relatively small differences in the natural environment, economic development level, and social conditions, sharing the same agricultural production combination. The agricultural land crops primarily include wheat, corn, peanuts, honeysuckle, and garlic, and the forest and fruit crops are predominantly chestnuts and peaches. The timing of various crop production activities is as follows: wheat planting is from early October to early June of the following year; corn and peanut planting is from early June to early October; garlic planting is from early October to the end of May of the following year; the honeysuckle planting time is from the beginning of August to the middle and early May of the following year; and the chestnut planting time is from the beginning of April to the end of September. Different crops are not compatible in terms of the planting time and agricultural land use, and peasant households must make reasonable choices in agricultural production to achieve the optimal agricultural production combination.

##### Resource Constraints

This study selected agricultural land, labor, and capital as the fundamental resource constraints. Regarding agricultural land constraints, according to the planting system in the study area, the maximum multiple cropping index was set to 2.0, with two crops a year as the norm and three crops in two years as a supplement, assuming that the soil texture was uniform. The upper limit of the area sown is the product of the agricultural land area and the maximum multiple cropping index. For capital constraints, the upper limit of the total capital input is the average value of the peasant households’ agricultural production costs (the agricultural production expenditure). For the labor constraints, the upper limit of the labor input is the sum of the workdays of the households’ agricultural labor force (i.e., the product of the number of agricultural laborers and the maximum annual workdays). In the context of the study area, the maximum workdays per agricultural laborer per year was set to 300.

##### Target Income

In this study, the agricultural income of each type of peasant household in the study area was defined as the target income of agricultural production, which was defined as the “normal” target income, and the minimum survival target for each type of peasant household in the study area was defined as the “safe” target income. The main source of income for the *FTPH* was agricultural production; thus, their survival goals should meet the needs of the agricultural production costs and household living expenses (i.e., the sum of the household agricultural production expenditure and household living expenses). Other types of peasant households had non-agricultural income sources; thus, their survival goals should meet the needs of the agricultural production costs (i.e., the household agricultural production expenses).

#### 3.4.2. Optimal Agricultural Income for Different Types of Peasant Households under Risk States

In this study, the parameters identified for the different types of peasant households were substituted into the Target MOTAD model to determine the peasant households’ optimal agricultural income and production combinations under different production targets (Table 5).

As seen in Table 4, the actual optimal agricultural income available to the *FTPH* under the two target income parameters was 16,335.82 CNY, higher than the current income (13,109.11 CNY), and none of them had production risks. The *FTPH* agricultural production combination was 2.63 mu of peanuts, 2.63 mu of garlic, 1.21 mu of honeysuckle, and 1.21 mu of chestnuts. The actual optimal agricultural income available to the *I PTPH* under both target income parameters was 20,160.52 CNY, which was higher than the current income (19,748.30 CNY). When targeting the normal target income, agricultural production had a certain risk (λ = 832.31), but there was no production risk for the safe target income. The *I PTPH* agricultural production combination was 1.00 mu of corn, 2.23 mu of peanuts, 2.23 mu of garlic, 2.23 mu of honeysuckle, and 2.23 mu of chestnuts. The actual optimal agricultural income available to the *II PTPH* under both target income parameters was 9948.57 CNY, higher than the current income (6628.85 CNY), and both were production risk-free, with an agricultural production combination of 0.99 mu of garlic, 0.99 mu of honeysuckle, and 2.33 mu of chestnuts. The actual optimal agricultural income available to the *NAPH* under both target incomes was 5364.73 CNY, higher than the current income (2695.57 CNY), and both were free of production risk, with an agricultural production combination of 0.33 mu of garlic, 0.33 mu of honeysuckle, and 2.00 mu of chestnuts.

#### 3.4.3. Optimal Allocation of Agricultural Input Factors for Different Types of at-Risk Peasant Households

To optimize the allocation of the agricultural input factors for different types of peasant households, the existing agricultural land input resources were fixed as a constraint. The capital and labor input levels of the different types of peasant households were gradually altered to determine the optimal allocation of the agricultural input factors for the different types of at-risk peasant households. The target incomes were set as the optimal income under the constant resource constraint of agricultural land (i.e., 16,335.82, 20,160.52, 9948.57, and 5364.73 CNY for the *FTPH*, *I PTPH*, *II PTPH*, and *NAPH*, respectively).

##### Optimal Allocation of Agricultural Input Factors for *FTPH*

The labor input of the *FTPH* was assumed to be constant at 579 workdays (Table 6). If the *FTPH* capital input was 6068.22 CNY, and the actual agricultural income available was the target income, with a risk value λ = 702.12 (Combination IV). If the capital input was lower than 6068.22 CNY, the real agricultural income would gradually decrease, the production risk would gradually increase, and the structure of the agricultural land use would tend to be simpler (Combinations I, II, and III). If the capital input was higher than 6068.22 CNY, the actual agricultural income would gradually increase, and the production risk would gradually decrease. Until the capital input increased to 6337 CNY, the actual agricultural income available was 16,753.84 CNY, which was higher than the target income, and the risk value decreased to λ = 671.80 (Combination V). If the capital input continued to increase above 6337 CNY, the actual agricultural income available would no longer increase, and the risk value would remain the same. Therefore, when the labor input of the *FTPH* remained unchanged, the optimal capital input was 6337 CNY, and the agricultural land-use structure at this time was 1.92 mu of peanuts, 1.92 mu of garlic, 1.92 mu of honeysuckle, and 1.92 mu of chestnuts (Combination V).

The capital input of the *FTPH* was assumed to be constant at 6337 CNY (Table 7). If the labor input was 579 workdays, and the actual agricultural income available was 16,573.84 CNY, with a risk value of λ = 839.01 (Combination II). If the labor input increased, the actual agricultural income, risk value, and agricultural land-use structure would remain the same (Combination I). If the labor input gradually decreased, the actual agricultural income showed a constant trend then decreasing until it reached 266 workdays, the optimal agricultural income available was 16,753.84 CNY, and the risk value and agricultural land-use structure remained the same (Combination IV). If the labor input continued to decrease below 266 workdays, the actual agricultural income available would gradually decrease, the risk value would gradually increase, and the agricultural land-use structure would change (Combination V). Therefore, the optimal labor input for the *FTPH* with a capital input of 6337.00 CNY was 266 workdays (Combination IV).

##### Optimal Allocation of Agricultural Input Factors for *I PTPH*

The labor input of the *I PTPH* was assumed to be constant at 615 workdays (Table 8). If the capital input was 8724.64 CNY, and the actual agricultural income available was the target income, with a risk value of λ = 997.19 (Combination IV). If the capital input was lower than 8724.64 CNY, the actual agricultural income showed a constant trend, then decreasing until reaching 7937.00 CNY, the actual agricultural income available was still equal to the target income, and the risk value remained the same (Combination III). If the capital input continued to decrease below 7937.00 CNY, the real agricultural income available would gradually decrease and the risk value would gradually increase (Combination I and Combination II). If the capital input was higher than 8724.64 CNY, the real agricultural income available would no longer increase, and the production risk and agricultural land-use structure would remain the same (Combination V). Therefore, when the labor input of the *I PTPH* remained unchanged and the optimal capital input was 7937.00 CNY, the structure of the agricultural land use at this time was 1.00 mu of corn, 2.23 mu of peanuts, 2.23 mu of garlic, 2.23 mu of honeysuckle, and 2.23 mu of chestnuts (Combination III).

The capital input of the *I PTPH* was assumed to be constant at 7937.00 CNY (Table 9). If the *I PTPH* labor input was 615 workdays, and the actual agricultural income available was 20,160.52 CNY, with a risk value λ = 997.19 (Combination II). If the labor input increased, the actual agricultural income available, risk value, and agricultural land-use structure would remain the same (Combination I). If the labor input decreased, the actual agricultural income showed a trend of constant and then decreasing until it decreased to 269 workdays, the optimal income obtainable would still be 20,160.52 CNY, and the risk value and agricultural land-use structure would remain the same (Combination IV). If the labor input continued to decrease, the actual agricultural income available would gradually decrease, the risk value would increase, and the structure of the agricultural land use would change (Combination V). Therefore, the optimal labor input was 269 workdays when the capital input of the *I PTPH* was 7937.00 CNY (Combination IV).

##### Optimal Allocation of Agricultural Input Factors for *II PTPH*

The labor input of the *II PTPH* was assumed to be constant at 594 workdays (Table 10). If the *II PTPH* capital input was 3300.86 CNY, and the actual agricultural income available was the target income, with a risk value of λ = 669.38 (Combination III). If the capital input was lower than 3300.86 CNY, the actual agricultural income available would gradually decrease, and the risk value would gradually increase (Combination I and Combination II). If the capital input was higher than 3300.86 CNY, the actual agricultural income available would gradually increase and the risk value would gradually decrease (Combination IV). When the capital input increased to 4047.00 CNY, the optimal agricultural income available was 11,932.89 CNY, which was higher than the target income, and the risk value decreased to 0 (Combination V). If the capital input continued to increase above 4047.00 CNY, the actual agricultural income available would no longer increase, and the risk value and agricultural land-use structure would remain unchanged. Therefore, when the labor input of the *II PTPH* was constant and the optimal capital input was 4047.00 CNY, the structure of the agricultural land use at this time was 1.43 mu of garlic, 1.43 mu of honeysuckle, and 1.89 mu of chestnut (Combination V).

The capital input of the *II PTPH* was assumed to be constant at 4047.00 CNY (Table 11). If the *II PTPH* labor input was 594.00 workdays, and the actual agricultural income available was 11,932.89 CNY, with a risk value λ = 962.42 (Combination II). If the labor input increased, the actual agricultural income available, risk value, and agricultural land-use structure would remain the same (Combination I). If the labor input decreased, the actual agricultural income available showed a constant trend, then decreasing until reaching 153 workdays, the optimal agricultural income available was 11,932.89 CNY, and the risk value and agricultural land-use structure remained the same (Combination IV). If the labor input continued to decrease, the actual agricultural income available would gradually decrease, the risk value would also increase, and the structure of the agricultural land use would change (Combination V). Therefore, the optimal labor input was 153 workdays when the capital input of the *II PTPH* was 4047.00 CNY (Combination IV).

##### Optimal Allocation of Agricultural Input Factors for *NAPH*

The labor input of the *NAPH* was assumed to be constant at 612 workdays (Table 12). If the *NAPH* capital input was 1708.45 CNY, and the actual agricultural income available was the target income, with a risk value of λ = 502.20 (Combination III). If the capital input was lower than 1708.45 CNY, the actual agricultural income available would gradually decrease and the risk value would gradually increase (Combination I and Combination II). If the capital input was higher than 1708.45 CNY, the actual agricultural income available would gradually increase and the risk value would gradually decrease (Combination IV). When the capital input increased to 2173.00 CNY, the optimal agricultural income available was 6600.17 CNY, which was higher than the target income, and λ = 0 (Combination V). If the capital input continued to increase above 2173.00 CNY, the actual agricultural income available would no longer increase, and the risk value and agricultural land-use structure would remain unchanged. Therefore, when the labor input of the *NAPH* remained unchanged and the optimal capital input was 2173.00 CNY, the structure of the agricultural land use at this time was 0.61 mu of garlic, 0.61 mu of honeysuckle, and 1.72 mu of chestnuts (Combination V).

The capital input of the *NAPH* was assumed to be constant at 2173.00 CNY (Table 13). If the *NAPH* labor input was 612 workdays, and the actual agricultural income available was 6600.17 CNY, with a risk value λ = 477.43 (Combination II). If the labor input increased, the actual agricultural income, risk value, and agricultural land-use structure would remain unchanged (Combination I). If the labor input decreased, the actual agricultural income showed a constant trend, then decreasing until reaching 99 workdays, the optimal agricultural income available was 6600.17 CNY, and the risk value and agricultural land-use structure remained the same (Combination IV). If the labor input continued to decrease, the actual agricultural income available would gradually decrease, the risk value would increase, and the structure of the agricultural land use would change (Combination V). Therefore, the optimal labor input was 99 workdays when the capital input of the *NAPH* was 2173.00 CNY (Combination IV).

#### 3.4.4. Optimal Allocation of Agricultural Input Factors and Production Combinations for Different Types of Peasant Households under Risk States

Based on the above optimization of the agricultural input factors and production combinations for the different types of peasant households, the optimal allocation of the agricultural input factors and the agricultural production combinations as well as the agricultural incomes for each type of peasant household under risk states were obtained (Table 14).

**Figure 3:**
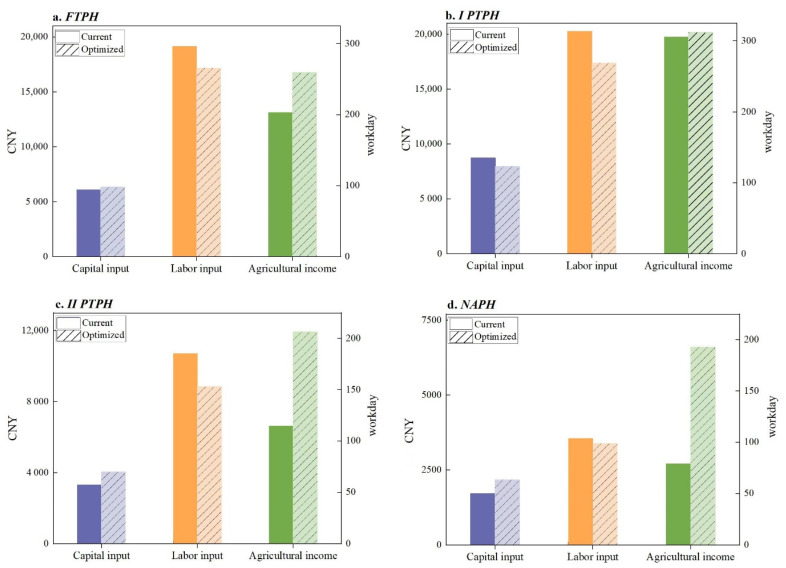
Comparison of agricultural inputs and incomes of different types of peasant households before and after optimization.

Comparing the current inputs with the optimal inputs of the different types of peasant households (Figure 3), it was found that all types of peasant households must continue to increase the capital inputs, except for the *I PTPH*, who had excess capital inputs which required a reduction. All the types of the peasant households had excess labor inputs and must streamline the labor inputs. In terms of the agricultural income, all the types of the peasant households experienced increased agricultural returns, with the highest increase for the *II PTPH* and the smallest increase for the *I PTPH*.

Specifically, after optimization, the *FTPH* must increase the capital input by 268.78 CNY and reduce the labor input by 30.32 workdays, and the actual agricultural income available will increase by 3644.73 CNY. The *I PTPH* must reduce the capital input by 787.64 CNY and reduce the labor input by 44.57 workdays, and the actual agricultural income available increases by 421.22 CNY. The *II PTPH* must increase the capital input by 746.14 CNY and reduce the labor input by 32.50 workdays, and the actual agricultural income available will increase by 5304.04 CNY. The *NAPH* must increase the capital input by 464.55 CNY and reduce the labor input by 4.66 workdays, and the actual agricultural income available will increase by 3904.60 CNY.

## 4. Discussion

### 4.1. Differences in Agricultural Input Levels among Different Types of Peasant Households

The agricultural input level of the *I PTPH* was higher than that of the *FTPH*, which was related to the differences in the basic household characteristics and business objectives. The *I PTPH* had a larger household size, a larger area of agricultural land, and a larger number of household agricultural laborers, and therefore higher levels of total labor input and total capital input, whereas the agricultural income of the *I PTPH* was the primary source of household income, accounting for 51.87%. To achieve higher agricultural income goals, many households chose to grow economic crops with complex daily management, such as garlic, peaches, and chestnuts. They preferred to increase the output of their agricultural land by increasing the labor and capital inputs per unit area. Some household members also worked outside the home to increase their income, easing the financial constraints on household agricultural production. The farmers would prioritize the acquisition of productive agricultural assets to increase the agricultural land output, resulting in higher levels of both household labor and capital inputs for the *I PTPH*. Some scholars’ research has supported this view, arguing that the appropriate part-time employment of peasant households would improve households’ agricultural investment capacity and risk resistance [51,52,53].

Although the *II PTPH* non-agricultural income was higher, the level of agricultural input was lower than that of the *FTPH* and *I PTPH*, primarily because the *II PTPH* business activities were non-agricultural, and the household labor force had to balance agricultural and non-agricultural production. Still, the incomes obtained from engaging in non-agricultural production were higher, so the *II PTPH* invested more time and capital into non-agricultural activities. However, the capital input of the *II PTPH* did not increase with the rise In non-agricultural income, which indicated that the *II PTPH* dependence on agriculture decreased, leading to decreasing labor and capital inputs rather than rising. Some scholars have demonstrated that non-agricultural employment reduced the input of labor, capital, and other factors per unit of land, causing changes in the original intensive farming patterns [54,55].

The family structure of the *NAPH* predominantly belonged to the “two-generation” type. The household labor forces faced the dual family pressure of children going to school and supporting the elderly, and the family labor forces were relatively young and had a relatively high level of education so were more inclined toward non-agricultural activities in resource allocation decisions and less motivated to invest in agricultural production. Subsequently, their labor and capital inputs were significantly reduced. The survey found that the vast majority of the *NAPH* chose to plant wheat, corn, and other grain crops, which were easy to manage and required less investment, resulting in less labor and capital input. Although the agricultural land area of the *NAPH* had declined, they still maintained a certain amount of agricultural land. This also indicated that the *NAPH* were affected by the idea of “leaving the countryside but not the land,” and they were more willing to regard agricultural land as livelihood security. Similarly, some scholars have asserted that the employment focus of peasant households with long-term employment would shift to non-agricultural activities, which would reduce the input in agricultural production [56,57,58].

A comparative analysis of the agricultural input characteristics of the different types of peasant households revealed that the *I PTPH* maintained higher levels of agricultural land, labor, and capital inputs, which were significantly higher than those of other types of peasant households. However, the input levels of the *II PTPH* and *NAPH*, with higher levels of part-time employment gradually decreasing, indicated that appropriate part-time employment can promote peasant households’ increase in effective inputs in agricultural production. In contrast, when the degree of part-time employment exceeded a certain threshold, the peasant households’ dependence on agriculture gradually decreased, tending to shift their time and capital to non-agricultural activities, and the labor and capital inputs in agricultural production may decline.

### 4.2. Optimization of Agricultural Inputs and Production Combinations for Different Types of Peasant Households

Due to resource and capacity constraints, peasant households face multiple risks in agricultural production. From the perspective of peasant households, crop yield and agricultural income are the two most concerning aspects. This study used the Target MOTAD model to analyze the optimization of agricultural inputs and production combinations of different types of peasant households in the Yimeng Mountain area, finding that different types of peasant households could choose the optimal production combinations according to risk preferences. Moreover, households could adjust agricultural input factors and production combinations, such as agricultural land, labor, and capital, to achieve expected target incomes. Consequently, this model can be widely used in the study of peasant households’ production decisions. The key to establishing the Target MOTAD model is to determine the target incomes. When analyzing the production decisions at the peasant household level, such target incomes can be accurately formulated based on household characteristics and income and expenditure budgets. At the same time, the optimal production combination under the tolerable risk level can be determined according to peasant households’ risk preferences. Some scholars have also developed risk-minimizing crop combinations and feasible farm plans based on the Target MOTAD model [10,24,37].

The results of this study also demonstrated that peasant households in the study area generally had challenges of low agricultural production efficiency and irrational agricultural input factors, such as excess labor input and insufficient capital input. This phenomenon is common in mountainous areas with backward economic development and poor land resources [59,60,61]. The results of this study can provide useful guidance for optimizing peasant households’ agricultural production and help peasant households rationally allocate agricultural input factors according to household resource endowments and target incomes and choose suitable agricultural production combinations to reduce agricultural risks and increase agricultural production income.

The uncertainties and risks associated with agriculture make agricultural production a complex process. If various types of risks are borne by peasant groups alone, it may lead to the misallocation of resources, thus reducing overall social welfare [30]. Most peasant households in China are self-sufficient smallholders who are more vulnerable to risks, such as uncertainties in agricultural investment returns and crop losses [37,61]. The results of this study indicated that risk has a vital role in peasant households’ production decisions and is related to peasant households’ choices and levels of inputs and outputs. Because agricultural production is a major source of income for peasant households, it is important for them to recognize and manage production risks [62,63,64]. Therefore, a timely approach to optimize the agricultural input and production combination can help peasant households to mitigate the negative effects of risks and to adopt adaptive measures. Local governments and related departments should focus on these research findings to develop reasonable agricultural risk management policies [65]. The government can increase the early warning and forecasting of weather and disaster information. They can also enhance the disclosure and dissemination of market information, increase technical training and advisory services for peasant households, and develop risk management tools (e.g., crop insurance and diversified and precautionary savings and credit), which can help peasant households adopt appropriate risk management strategies for navigating various uncertainties and risks in agriculture.

Although this study is limited to the Yimeng Mountain area in China, the findings may have broader implications that can be extended to other regions of the developing world. In general, these findings may help other regions and developing countries to strategically manage risks and advance sustainable agricultural development.

### 4.3. Limitations

In this study, the research model and findings presented can be used to optimize the analysis of farms in other different regions of China. However, the Target MOTAD model proposed in this paper contains certain limitations related to the assumptions, such as in the process of optimizing peasant households’ agricultural production combination and agricultural land input factors, the quality of agricultural land was assumed to be homogeneous, with the same productive capacity, and this assumption inevitably differed from the actual circumstances in the study area, where peasant households had contracted agricultural lands of different quality and plot sizes. Therefore, in future research, we have the possibility to develop an integrated tool to optimize agricultural production activities on various types of farms. At the same time, due to uncontrollable natural conditions and asymmetric market information, peasant households often face certain risks when making critical production decisions. This study only selected data on the fluctuation of the main agricultural products’ returns in Shandong Province in the past five years as the basis for simulating peasant households’ agricultural production decision-making behavior, and this was not comprehensive enough to capture the trend of risk fluctuation. Therefore, future research could further integrate a suitability evaluation of agricultural land to improve the agricultural production combinations and allocation structure of agricultural input factors and select more extended time-series data to reflect market fluctuation conditions.

## 5. Conclusions

This study used peasant households in two typical counties in the Yimeng Mountain area of northern China as the sample areas. Based on the survey data from 262 peasant households, the characteristics of the agricultural land input of different types of peasant households were analyzed. The Target MOTAD model was applied to optimize the agricultural input factors and agricultural production combinations of different types of peasant households under risk states. The conclusions are as follows:

(1) There were significant differences in the agricultural land, labor, and capital inputs among the different types of peasant households. In terms of the agricultural land input, the agricultural land scale of each type of peasant household was generally small, and the agricultural land fragmentation was severe, among which the highest for the *I PTPH* was 4.95 mu of agricultural land and 8.6 plots. The labor input per unit area of the *FTPH* was the highest, at 75.02 workdays/mu, while the total labor input of the *I PTPH* was the highest, at 313.57 workdays. In terms of the capital input, the sum of the yield-increasing input per unit area was much higher than the labor-saving input, among which the total capital input of the *I PTPH* was the highest at 8724.64 CNY. Overall, as the degree of part-time employment increased, the agricultural input level of each type of peasant household showed an inverted U-shaped trend of first increasing and then decreasing, namely *I PTPH* > *FTPH* > *II PTPH* > *NAPH*.
(2) The current agricultural inputs and production combinations of the different types of peasant households had room for improvement. Target incomes cannot be achieved at the current level of agricultural inputs and must be obtained by adjusting the agricultural inputs and optimizing production combinations. The *FTPH* must increase the capital input by 268.78 CNY and reduce the labor input by 30.32 workdays, which could increase the actual agricultural income by 3644.73 CNY. The *I PTPH* must reduce the capital input by 787.64 CNY and reduce the labor input by 44.57 workdays, which could increase the actual agricultural income by 421.22 CNY. The *II PTPH* must increase the capital input by 746.14 CNY and reduce the labor input by 32.50 workdays, which could increase the actual agricultural income by 5304.04 CNY. The *NAPH* must increase the capital input by 464.55 CNY and reduce the labor input by 4.66 workdays, which could increase the actual agricultural income by 3904.60 CNY. In general, with the continuous optimization of agricultural inputs and production combinations, the agricultural incomes could be improved as the cultivation of economic crops, such as peanuts, garlic, honeysuckle, and chestnuts, gradually replace grain crops, such as wheat and corn.

## Figures and Tables

**Table 1:** Criteria for classifying peasant household types.

Type of Peasant Household	Main Livelihood	Allocation Method of Agricultural and Sideline Products	Main Sources of Household Income	Household Income Structure
*FTPH*	Agriculture	Mostly for market allocation, a few for self-production and self-sales	Agricultural income, government subsidies	*AIP >* 95%
*I PTPH*	Agriculture, non-agriculture	Mostly for market allocation, a few for self-production and self-sales	Agricultural income, non-agricultural income	50% < *AIP* ≤ 95%
*II PTPH*	Non-agriculture, agriculture	Partly for market allocation, a few for self-production and self-sales	Non-agricultural income, agricultural income	50% < *NAIP* ≤ 95%
*NAPH*	Non-agriculture	Mostly for self-production and self-sales, a few for market allocation	Non-agricultural income	*NAIP >* 95%

Note: AIP represents the proportion of agricultural income in total household income; NAIP represents the proportion of non-agricultural income in total household income.

**Table 2:** Description of selected variables for Target MOTAD model.

Category	Selected Variables	Unit	Description of Variables
Resource constraints	Multiple cropping index	/	Average number of crops grown on agricultural land in a year
	Household agricultural land area	mu	Total area of agricultural land input agricultural production
	Crop-planted area	mu	Planted area for each crop
	Crop workday input	day/mu	Unit area workday input for each crop
	Crop capital input	CNY/mu	Unit area capital input for each crop
	Household agricultural labor force	Person	The sum of labor force input in agricultural production
	Workday of agricultural labor force	day/year	Per agricultural labor force input workday
Target income	Household agricultural production expenditure	CNY/year	Total capital input in agricultural production
	Household living expenses	CNY/year	Total capital input in daily living consumption
	Household agricultural income	CNY/year	Total income from agricultural production

Note: Mu is a unit of land area in China, 1 mu = 0.0667 ha; the mean conversion rate for the years covered by the survey is 6.6 CNY: 1 USD.

**Table 3:** Basic household characteristics of different types of peasant households.

Household Characteristics Indicators	*FTPH*	*I PTPH*	*II PTPH*	*NAPH*
Household size (person)	2.38	4.15	4.23	4.30
Household labor force (person)	1.93	3.2	3.12	3.39
Of which: agricultural labor force (person)	1.93	2.05	1.98	2.04
Age of agricultural labor force (year)	58.68	53.6	52.35	53.06
The literacy level of agricultural labor force	1.71	2.61	2.13	2.31
Per capita agricultural land area (mu)	1.61	1.19	0.78	0.54
Total household income (CNY/year)	14,498.84	38,071.85	41,215.91	57,670.35
Household non-agricultural income (CNY/year)	0.00	17,841.30	34,057.79	54,869.57
Household agricultural income (CNY/year)	13,109.11	19,748.30	6628.85	2695.57
Household non-labor income (CNY/year)	1389.73	482.25	529.27	105.22
Household income structure (%)	90.41	51.87	16.08	4.67
Total household expenditure (CNY/year)	12,655.50	25,054.64	22,097.62	25,273.67
Household agricultural production expenditure (CNY/year)	6068.22	8724.64	3300.86	1708.45
Household living expenses (CNY/year)	6587.28	16,330.00	18,796.76	23,565.22
Household expenditure structure (%)	47.95	34.82	14.94	6.76

Note: Among the indicators measuring the literacy level of agricultural labor force, illiteracy = 1; primary school = 2; junior school = 3; senior school = 4; college or above = 5. Household income structure is the ratio of agricultural income to total income. Household expenditure structure is the ratio of agricultural production expenditure to total expenditure.

**Table 4:** Agricultural inputs of different types of peasant households.

Types of Peasant Households	Agricultural Land Input		Labor Input		Yield-Increasing Input				Labor-Saving Input	Total Capital Input (CNY)
	Agricultural Land Area (mu)	Number of Agricultural Land Plots (Piece)	Labor Input per Unit Area (Workday/mu)	Total Labor Input (Workday)	Seed (CNY/mu)	Pesticide (CNY/mu)	Fertilizer (CNY/mu)	Agricultural Film (CNY/mu)	Mechanical Power (CNY/mu)	
*FTPH*	3.84	4.20	75.02	296.32	220.96	380.37	161.94	44.57	125.42	6068.22
*I PTPH*	4.95	8.60	63.35	313.57	198.31	482.24	318.66	25.82	167.61	8724.64
*II PTPH*	3.32	4.77	55.87	185.50	130.62	348.10	119.10	22.99	115.64	3300.86
*NAPH*	2.33	4.38	44.49	103.66	85.35	279.45	42.63	23.90	114.59	1708.45
Total	3.39	5.01	53.55	204.09	140.07	317.86	98.98	27.06	118.70	4055.91

**Table 5:** The optimal agricultural incomes and production combinations for different types of peasant households.

	Target Income	*FTPH*		*I PTPH*		*II PTPH*		*NAPH*	
Production Activities		Normal Target Income	Safe Target Income	Normal Target Income	Safe Target Income	Normal Target Income	Safe Target Income	Normal Target Income	Safe Target Income
Agricultural income (CNY)		16,335.82	16,335.82	20,160.52	20,160.52	9948.57	9948.57	5364.73	5364.73
Risk value (λ)		0.00	0.00	832.31 *	0.00	0.00	0.00	0.00	0.00
Wheat (mu)		0.00	0.00	0.00	0.00	0.00	0.00	0.00	0.00
Corn (mu)		0.00	0.00	1.00	1.00	0.00	0.00	0.00	0.00
Peanut (mu)		2.63	2.63	2.23	2.23	0.00	0.00	0.00	0.00
Garlic (mu)		2.63	2.63	2.23	2.23	0.99	0.99	0.33	0.33
Honeysuckle (mu)		1.21	1.21	2.23	2.23	0.99	0.99	0.33	0.33
Chestnut (mu)		1.21	1.21	2.23	2.23	2.33	2.33	2.00	2.00

Note: * denotes the maximum value of λ when the Target MOTAD model elicits an optimal solution.

**Table 6:** Optimization of capital input for *FTPH*.

Production Activities	Combination I	Combination II	Combination III	Combination IV	Combination V
Labor input (workday)	579.00	579.00	579.00	579.00	579.00
Capital input (CNY)	5000.00	5500.00	6000.00	6068.22	≤6337.00
Actual agricultural income (CNY)	14,553.02	15,428.83	16,229.38	16,335.82	16,753.84
Risk value (λ)	1218.38 *	822.25 *	709.84 *	702.12 *	671.80 *
Wheat (mu)	0.00	0.00	0.00	0.00	0.00
Corn (mu)	0.00	0.00	0.00	0.00	0.00
Peanut (mu)	2.67	3.63	2.8	2.63	1.92
Garlic (mu)	3.84	3.84	2.8	2.63	1.92
Honeysuckle (mu)	0.00	0.00	1.04	1.21	1.92
Chestnut (mu)	0.00	0.00	1.04	1.21	1.92

Note: * denotes the maximum value of λ when the Target MOTAD model elicits an optimal solution.

**Table 7:** Optimization of labor input for *FTPH*.

Production Activities	Combination I	Combination II	Combination III	Combination IV	Combination V
Capital input (CNY)	6337.00	6337.00	6337.00	6337.00	6337.00
Labor input (workday)	>579.00	579.00	296.32	266.00	240.00
Actual agricultural income (CNY)	16,753.84	16,753.84	16,753.84	16,753.84	16,035.72
Risk value (λ)	839.01 *	839.01 *	839.01 *	839.01 *	891.10 *
Wheat (mu)	0.00	0.00	0.00	0.00	0.00
Corn (mu)	0.00	0.00	0.00	0.00	0.00
Peanut (mu)	1.92	1.92	1.92	1.92	3.13
Garlic (mu)	1.92	1.92	1.92	1.92	3.13
Honeysuckle (mu)	1.92	1.92	1.92	1.92	0.71
Chestnut (mu)	1.92	1.92	1.92	1.92	0.71

Note: * denotes the maximum value of λ when the Target MOTAD model elicits an optimal solution.

**Table 8:** Optimization of capital input for *I PTPH*.

Production Activities	Combination I	Combination II	Combination III	Combination IV	Combination V
Labor input (workday)	615.00	615.00	615.00	615.00	615.00
Capital input (CNY)	7000.00	7500.00	7937.00	8724.64	≤9000.00
Actual agricultural income (CNY)	19,158	19,718.78	20,160.52	20,160.52	20,160.52
Risk value (λ)	1115.17 *	1029.24 *	997.19 *	997.19 *	997.19 *
Wheat (mu)	0.00	0.00	0.00	0.00	0.00
Corn (mu)	0.79	1.00	1.00	1.00	1.00
Peanut (mu)	3.66	2.97	2.23	2.23	2.23
Garlic (mu)	3.66	2.97	2.23	2.23	2.23
Honeysuckle (mu)	0.79	1.48	2.23	2.23	2.23
Chestnut (mu)	0.79	1.48	2.23	2.23	2.23

Note: * denotes the maximum value of λ when the Target MOTAD model elicits an optimal solution.

**Table 9:** Optimization of labor input for *I PTPH*.

Production Activities	Combination I	Combination II	Combination III	Combination IV	Combination V
Capital input (CNY)	7937.00	7937.00	7937.00	7937.00	7937.00
Labor input (workday)	>615.00	615.00	313.57	269.00	260.00
Actual agricultural income (CNY)	20,160.52	20,160.52	20,160.52	20,160.52	19,945.51
Risk value (λ)	997.19 *	997.19 *	997.19 *	997.19 *	1012.79 *
Wheat (mu)	0.00	0.00	0.00	0.00	0.00
Corn (mu)	1.00	1.00	1.00	1.00	1.00
Peanut (mu)	2.23	2.23	2.23	2.23	2.59
Garlic (mu)	2.23	2.23	2.23	2.23	2.59
Honeysuckle (mu)	2.23	2.23	2.23	2.23	1.86
Chestnut (mu)	2.23	2.23	2.23	2.23	1.86

Note: * denotes the maximum value of λ when the Target MOTAD model elicits an optimal solution.

**Table 10:** Optimization of capital input for *II PTPH*.

Production Activities	Combination I	Combination II	Combination III	Combination IV	Combination V
Labor input (workday)	594.00	594.00	594.00	594.00	594.00
Capital input (CNY)	2500.00	3000.00	3300.86	3500.00	≤4047.00
Actual agricultural income (CNY)	7818.74	9148.46	9948.57	10,478.17	11,932.89
Risk value (λ)	2693.04 *	1165.49 *	669.38 *	452.24 *	0.00 *
Wheat (mu)	0.00	0.00	0.00	0.00	0.00
Corn (mu)	0.00	0.00	0.00	0.00	0.00
Peanut (mu)	0.00	0.00	0.00	0.00	0.00
Garlic (mu)	0.51	0.81	0.99	1.10	1.43
Honeysuckle (mu)	0.51	0.81	0.99	1.10	1.43
Chestnut (mu)	2.81	2.51	2.33	2.22	1.89

Note: * denotes the maximum value of λ when the Target MOTAD model elicits an optimal solution.

**Table 11:** Optimization of labor input for *II PTPH*.

Production activities	Combination I	Combination II	Combination III	Combination IV	Combination V
Capital input (CNY)	4047.00	4047.00	4047.00	4047.00	4047.00
Labor input (workday)	>594.00	594.00	185.50	153.00	130.00
Actual agricultural income (CNY)	11,932.89	11,932.89	11,932.89	11,932.89	11,896.44
Risk value (λ)	962.42 *	962.42 *	962.42 *	962.42 *	976.11 *
Wheat (mu)	0.00	0.00	0.00	0.00	0.00
Corn (mu)	0.00	0.00	0.00	0.00	0.00
Peanut (mu)	0.00	0.00	0.00	0.00	0.00
Garlic (mu)	1.43	1.43	1.43	1.43	1.14
Honeysuckle (mu)	1.43	1.43	1.43	1.43	2.08
Chestnut (mu)	1.89	1.89	1.89	1.89	1.24

Note: * denotes the maximum value of λ when the Target MOTAD model elicits an optimal solution.

**Table 12:** Optimization of capital input for *NAPH*.

Production Activities	Combination I	Combination II	Combination III	Combination IV	Combination V
Labor input (workday)	612.00	612.00	612.00	612.00	612.00
Capital input (CNY)	1500.00	1600.00	1708.45	2000.00	≤2173.00
Actual agricultural income (CNY)	4810.37	5076.32	5364.73	6140.09	6600.17
Risk value (λ)	845.93 *	681.03 *	502.20 *	152.94 *	0.00 *
Wheat (mu)	0.00	0.00	0.00	0.00	0.00
Corn (mu)	0.00	0.00	0.00	0.00	0.00
Peanut (mu)	0.00	0.00	0.00	0.00	0.00
Garlic (mu)	0.21	0.27	0.33	0.51	0.61
Honeysuckle (mu)	0.21	0.27	0.33	0.51	0.61
Chestnut (mu)	2.12	2.06	2.00	1.82	1.72

Note: * denotes the maximum value of λ when the Target MOTAD model elicits an optimal solution.

**Table 13:** Optimization of labor input for *NAPH*.

Production Activities	Combination I	Combination II	Combination III	Combination IV	Combination V
Capital input (CNY)	2173.00	2173.00	2173.00	2173.00	2173.00
Labor input (workday)	>612.00	612.00	103.66	99.00	90.00
Actual agricultural income (CNY)	6600.17	6600.17	6600.17	6600.17	6574.58
Risk value (λ)	477.43 *	477.43 *	477.43 *	477.43 *	532.42 *
Wheat (mu)	0.00	0.00	0.00	0.00	0.00
Corn (mu)	0.00	0.00	0.00	0.00	0.00
Peanut (mu)	0.00	0.00	0.00	0.00	0.00
Garlic (mu)	0.61	0.61	0.61	0.61	0.4
Honeysuckle (mu)	0.61	0.61	0.61	0.61	1.07
Chestnut (mu)	1.72	1.72	1.72	1.72	1.26

Note: * denotes the maximum value of λ when the Target MOTAD model elicits an optimal solution.

**Table 14:** Optimal allocation of agricultural input factors and production combinations for different types of peasant households.

Production Activities	Combination I	Combination II	Combination III	Combination IV	Combination V
Capital input (CNY)	6337.00	7937.00	4047.00	2173.00	6337.00
Labor input (workday)	266.00	269.00	153.00	99.00	266.00
Optimal agricultural income (CNY)	16,753.84	20,160.52	11,932.89	6600.17	16,753.84
Risk value (λ)	839.01 *	997.19 *	962.42 *	477.43 *	839.01 *
Wheat (mu)	0.00	0.00	0.00	0.00	0.00
Corn (mu)	0.00	1.00	0.00	0.00	0.00
Peanut (mu)	1.92	2.23	0.00	0.00	1.92
Garlic (mu)	1.92	2.23	1.43	0.61	1.92
Honeysuckle (mu)	1.92	2.23	1.43	0.61	1.92
Chestnut (mu)	1.92	2.23	1.89	1.72	1.92

Note: * denotes the maximum value of λ when the Target MOTAD model elicits an optimal solution.

## Data Availability

The data used to support the findings of this study are available from the corresponding author upon request.
